# Optimal Design and Operation for a No-Moving-Parts-Valve (NMPV) Micro-Pump with a Diffuser Width of 500 μm

**DOI:** 10.3390/s90503666

**Published:** 2009-05-15

**Authors:** Chin-Tsan Wang, Tzong-Shyng Leu, Jia-Ming Sun

**Affiliations:** 1 Department of Mechanical and Electro-Mechanical Engineering, National I Lan University / 1, Sec. 1, Shen-Lung Road, I-Lan, 26047, Taiwan; 2 Department of Aeronautics and Astronautics, National Cheng Kung University / 1, Ta-Hsueh Road, Tainan 70101, Taiwan; E-Mails: tsleu@mail.ncku.edu.tw; s0925.jms@msa.hinet.net

**Keywords:** no-moving-parts-valve (NMPV), expansion valve angle, net flow

## Abstract

A no-moving-parts-valve (NMPV) with a diffuser width of D = 500 microns was investigated in this study by numerical simulations at Reynolds numbers, Re_D_, ranging from 20 to 75, and expansion valve angles ranging from 30° < θ_1_ < 57° and 110° < θ_2_ < 120°. The *D^p^,_i_* value, 1.02 < *D^p^,_i_* < 1.14, is larger within the proposed range of the expansion valve angles. A flow channel structure with a depth of 500 micron is manufactured using yellow light lithography in this study. From prior analyses and experiments, it is found that piezoelectric films work better at a buzz driving frequency of *f* < 30Hz and the best operating frequency is at a driving frequency of *f* = 10Hz because it produces the largest net flow. In addition, the expansion angles θ_1_ = 30° and θ_2_ = 120° are the best expansion angles because they produce the largest net flow. These related results are very helpful for the actual design of no-moving-parts-valve micro-pump.

## 1. Introduction

In order to satisfy requirements in biomedical sample applications or reagent (single or mixing) applications, a micro-flow system requires: (1) a micro-flow driving element (2) a micro control valve (3) a micro-mixer. According to Ngyuen *et al.* [1] and Shoji and Esashi [2], micro-pumps can be divided into: (1) mechanical pumps with moving components. (2) non-mechanical pumps with no moving components. Examples for moving components are pistons or thin films etc. and no moving components are mainly electrophoresis or electro-osmosis devices as referred in Lee [3] or devices using an external high-pressure gas source to drive fluid. Therefore, non-mechanical pumps with no moving parts possess the advantage of being capable of avoiding mechanical wear and tear generated by moving components like pistons, etc., but the disadvantage is that there are limitations on the types of liquid that can be driven.

The micro-valve part can be divided mainly into two categories based on the actuating mechanism: (1) mechanical valves with moving components (active micro-valves) and (2) non-mechanical valves with no moving components (passive micro-valve). Their difference lies in that the active micro-valve has an actuator while the passive micro-valve does not. The kinds of active micro-valve commonly used are: electromagnetic [3], piezoelectric [4], shape memory alloy [5] and bias spring, electrostatic [6], thermopneumatic [7] and bi-metallic [8].

Whether they are active or passive, they all possess moving components. As a result, they all require complex and high cost production processes. More importantly, when the fluid used is not a pure liquid but a mixture of liquid and particles, the valve is easily damaged during opening and closing. Also, it is prone to the occurrence of congestion of fluid molecules at the location of valve hinge or at the time of opening or closing, while passive micro-valves have no such problems.

Among all the types of passive valve, the most common one is a diffuser valve, which was first proposed by Tesla [9]. The working principle of the expansion valve is to explore the asymmetric effect of a specific flow channel to induce uneven resistance for fluid flowing in different directions. Such resistance difference can result in different flow rates, i.e. because of the flow rate difference between the forward and reversed directions under a specific pressure difference; it will result in a net flow in one direction and produce an effect similar to a one-way valve [9]. The micro-pump proposed in 1993 by Stemme *et al.* [10] installs one expansion valve component each at both exit ends. Usually, the expansion angle is less than 20 degrees. Because the resistance will be smaller in the expanding direction of the flow channel, the reversed flow will form a nozzle effect due to the narrowing of the flow channel and result in a relatively larger resistance. As shown in the expansion valve [11,12] proposed in 1994, the difference lies in the substantial increase of the open angle of the expansion angle to 54.7 degrees. However, it still utilizes the same principle to generate the one-way valve effect.

Olsson *et al.* [13] utilized simulation and experimental methods to explore the pressure drop effect of the valve and also derives a series of empirical formulas for no-moving-parts micro-pump [14]. Recently, no-moving-parts-valve micro-pumps also underwent a lot of changes. Yang *et al.* [15] devised an obstacle in the flow channel during the manufacturing process of the flow channel, which results in an asymmetrical flow field to achieve the function of the valve. In 2002, Schluter *et al.* [16] devised a special micro-structure in the micro-flow channel, which is capable of reducing the flow resistance in one direction out of the two. It is further matched with an actuator to achieve the effect of driving fluid. In summary, these related papers mentioned above are all focus on the research to improve the driving power of NMPVs. The analyses performed in this study aim mainly at the optimization of micro-flow channels for an NMPV with a diffuser width, D = 500 μm.

## 2. Problem Research and Discussion

The steady and unsteady boundary conditions in this study are set up as follows: First, steady state is used as the analysis condition to study five flow speed conditions for an NMPV: a series of different expansion valve angles and Reynolds numbers, Re_D_ = 75, 50, 20, 10, 5. When fluid flows through the valve, the fluid flowing in the forward direction experiences an unequal resistance from that flowing in the reverse direction, causing a different degree of pressure drop at the entrance/exit ends of the flow channel; a net pressure is produced as a result. The greater the pressure drop is, the larger the bipolarity of the valve; otherwise stated, the stronger the uni-directional property of the valve.

During the analysis process, Reynolds number is an important fluid parameter. Its definition is shown in Equation (1). Here, *ρ* is the density of the working fluid, *U*_ave_ is the characteristic speed (the average speed at the nozzle mouthpiece), *D* is the characteristic width (the width of the valve nozzle), and *μ* is the coefficient of viscosity of the liquid.

(1) ReD=ρ⋅Uave⋅Dμ

In the analysis, the “Forward Direction” is taken as the same direction as the positive X axis of the coordinates; otherwise, “Reversed Direction”. *D^p^,_i_* in name of mass flow rate addressed by Anders Olsson [14] is defined as the bi-polarity of the pressure drop valve at the entrance/exit ends of the flow channel and its definition is shown in Equation (2):

(2) $$D_{p}^{i}=\frac{p_{r}}{\Delta p_{f}}$$

After the steady state analysis of NMPV, the NMPV designed is used in the NMPV micro-pump in this study. Furthermore, in order to make the results of the steady state simulation more useful, three-dimensional unsteady state NMPV micro-pump system simulation is also undertaken in this study to study the pressure damage on the upper/lower wall of a micro-pipe in a NMPV micro-pump system. Additionally, the performance differences between two different designs is compared, fixing the expansion mouthpiece at 500 μm geometrically and comparing two different designs with expansion angles of θ_1_ = 20°, θ_2_ = 90°, and θ_1_ = 30°, θ_2_ = 120°, respectively.

Finally, experiments are carried out using the MEMS technology and SU-8 thick film photoresist to produce a NMPV micro-pump system master with a high aspect ratio. Flow channels for the NMPV micro-pump system are produced using PDMS mold and matched with a PZT actuator to form a NMPV micro-pump.

## 3. Numerical Experimentation Methods

**Figure 1.**
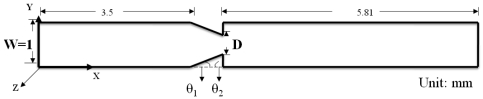
Schematic of the micro-channel geometry.

A numerical analysis software CFDRC 2009 was used as the simulation method in this study to research the effectiveness of NMPV under steady state fluid field conditions. The total length of NMPV under study is 10 mm; the main flow channel is set to 1 mm and uses an expansion mouthpiece or a nozzle mouthpiece as its main structure. The width of the expansion mouthpiece is designated as D and the expansion angles are θ1 and θ2, respectively, as shown in Figure 1. Structured Grid is used as the main method for the simulation analysis. It uses a finite element method to carry out the grid generation and calculate solutions based on continuous and momentum equations with matching boundary conditions.

The Structured Grid method was selected in consideration of the accuracy of the simulation analysis in this study. Also, in order to make up for the triangular cusp problem derived from the model after partition, additional trilateral geometric grids are established to effectively solve the model cusp problems during numerical calculations. 2D grid test runs were carried out before the research and it was found that a good total grid number to use was N = 15,561.

Water is used as the flow modeling object in this research. At room temperature (300 K) and one atmosphere pressure of the working environment, its relative density ρ_ref_ is 998.2 kg/m^3^ and its liquid viscosity coefficient μ_ref_ is 1.02×10^-3^ N s/m^2^.

**Figure 2.**
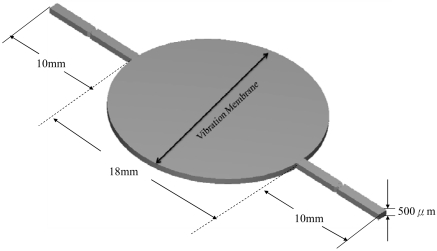
Three dimensional schematics of the NMPV micro-pump system.

A micro-actuator was also used as the pressure source in the three dimensional flow field and one atmosphere pressure at the entrance/exit on both ends of the micro-tube flow channel is used as the boundary condition. One NMPV component is placed at each side of the actuator, respectively, as shown in Figure 2. Grid tests are carried out on the NMPV micro-pump system to take into account the influence on the flow field from the upper/lower wall; the total grid number is found to be N = 218,000.

## 4. Results and Discussion

In the literature, all different expansion valve angles studied by Gerlach *et al.* [11] are able to generate a one-way valve effect. Therefore, in this study the expansion angles of an expansion valve with a diffuser width, D = 500 μm, is varied to conduct a series of investigations to find out the best design angles and operating conditions for the expansion valve.

In order to understand the relationship between the expansion angles (θ_1_, θ_2_) and *D^p^,_i_* better, analyses using optimization theory were carried out in this study: First, the boundary conditions are set to 20° < θ_1_ < 80° and 30° < θ_2_ < 140° and simulation nodes are generated using Design of Experiments (DOE) [17]. Then, Response Surface Modeling (RSM) [18] is utilized to find out the best recommended range. At the beginning of optimization, ten samples of (θ_1_, θ_2_) boundary conditions were selected arbitrarily as initial conditions for producing the D*^p^,_i_* for representing the amount of mass flow rate. Then, three samples were added to predict the D*^p^,_i_* for each cycle and one of Reynolds number Re_D_. A total of 10 cycles were applied and tested for the studied cases of Re_D_. Hence, in this study forty calculations were conducted to determine a response surface for finding the optimal (θ_1_, θ_2_, D*^p^,_i_*).

**Figure 3.**
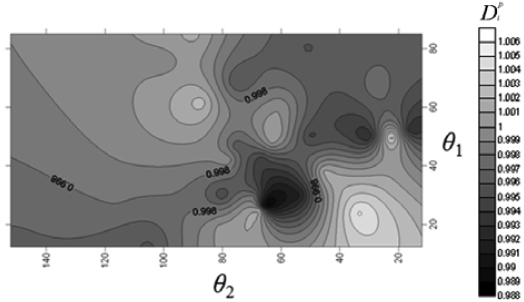
Chart for the relationship between θ_1_, θ_2_ and D*^p^,_i_* at Re_D_ = 5.

**Figure 4.**
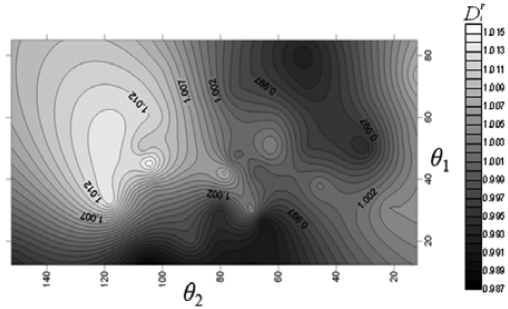
Chart for the relationship between θ_1_, θ_2_ and D*^p^,_i_* at Re_D_ = 20.

First, D = 500 μm is fixed for the NMPV to investigate the relationship between different flow channel speeds and the angles of the expansion valve. From Figure 3, it is found that D*^p^,_i_* exhibits a relatively irregular distribution at Reynolds number, Re_D_ = 5. When it is increased to Re_D_ = 20, it shows that there exists a larger D*^p^,_i_* area on the contour line map from Figure 4. Similarly, there also exists a larger D*^p^,_i_* area in the same direction at Re_D_ = 50 and Re_D_ = 75.

**Figure 5.**
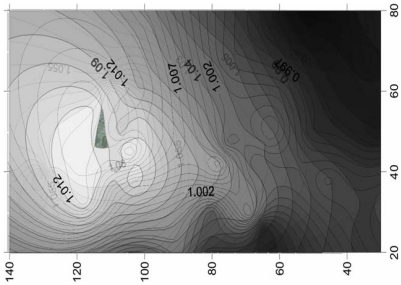
Chart for the relationship between θ_1_, θ_2_ and *D^p^,_i_* with D = 500 μm and 20 < Re_D_ < 75.

The results at Re_D_ = 20, Re_D_ = 50 and Re_D_ = 75 are overlapped to find a better common area through image processing in Figure 5. The region within 20 < Re_D_ < 75, 30° < *θ*_1_ < 57° and 110° < *θ_2_* < 120° is the recommended area for the expansion valve angles with 1.02 < D*^p^,_i_* < 1.14. The optimal region between the θ_1_, θ_2_ and D*^p^,_i_* for D = 500 μm at 20 < Re_D_ < 75, also confirmed by a numerical analysis software CFDRC version 2009 whose numerical accuracy of 10^-5^ makes it suitable for physical analysis.

In order to investigate real NMPV micro-pump systems and take into consideration the pressure damage to the upper/lower wall of the micro-pipe, a 3D NMPV micro-pump is built and the performance difference between two designs is also compared, i.e. fixing the expansion mouthpiece at 500 μm geometrically and comparing two different designs with the expansion angles of θ_1_ = 20°, θ_2_ = 90° and θ_1_ = 30°, θ_2_ = 120°.

**Figure 6.**
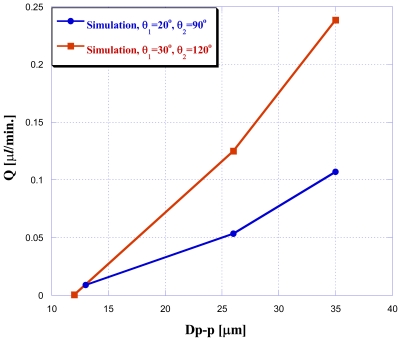
Chart of different driving pressures with θ_1_ = 20°, θ_2_ = 90° and θ_1_ = 30°, θ_2_ = 120°.

The vibration frequency was fixed at *f* = 10 Hz in this study to investigate the effect of different amplitude values ranging from 10 μm peak to peak to 35 μm peak to peak (Dp-p). The analysis results are shown in Figure 6: when doing three-dimensional NMPV micro-pump system simulations, design-optimized expansion valves have a better driving performance under the same driving conditions. Furthermore, the larger the driving amplitude is, the better the performance.

## 5. Manufacturing Processes and Experimental Equipments

**Figure 7.**
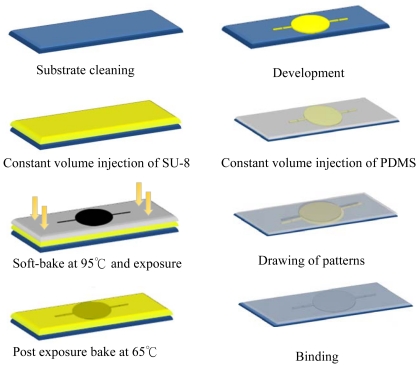
Diagrams for the manufacturing processes for the flow channel of the NMPV micro-pump system.

The considerations for manufacturing experimental micro-pump system were as follows: in order to reduce the pressure damage on the upper/lower wall inside micro-channel and take into consideration the actual manufacturing processes, the depth of the flow channel is chosen to be 500 μm. The experimental micro-pump system can be produced successfully using a yellow light lithography manufacturing process. First, thick-film SU-8 50 photoresist is used to produce a micro-pipe, which is a master structure with a depth of 500 μm made of SU-8. Then, the PDMS mold method is used to produce the micro-pipes for the NMPV micro-pump system. Figure 7 shows the manufacturing process.

Because a pipe shaped flow channel with high depth had to be manufactured in this study, the spin coating method, which distributes SU-8 photoresist on the substrate, cannot be used. Therefore, the volume for the expected depth is calculated beforehand after referencing publication [19]. When the SU-8 photoresist undergoes the soft bake process, its total volume will be reduced by 20% due to loss of water. However, the total volume required will be made up through injecting photoresist to the substrate. Soft bake time is the most important parameter for the manufacturing of this deep SU-8 structure.

**Figure 8.**
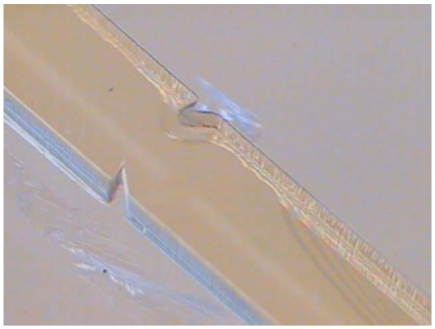
Drawing of the SU-8 structure with a height of 500 μm.

In the first phase, temperature is raised to 95°. The soft bake time is 3 hr when the photoresist thickness is 500 μm. In the second phase, it is cooled to room temperature after 30 minutes of soft bake at a temperature of 50 °C. Because the amount of exposure energy for SU-8 depends on the kind of material used for the substrate, glass is chosen was the substrate in this study. When the thickness of the photoresist is 500 μm, the energy required is 1,139.71 (Mj/cm^2^). Figure 8 shows the SU-8 structure with a height of 500 μm which was manufactured successfully using this process.

**Figure 9.**
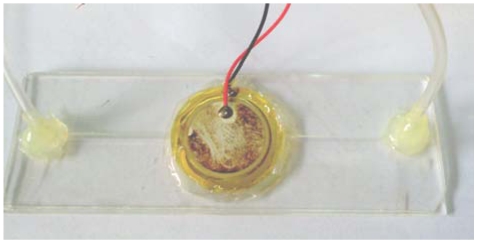
Drawing of the NMPV micro-pump system.

**Figure 10.**
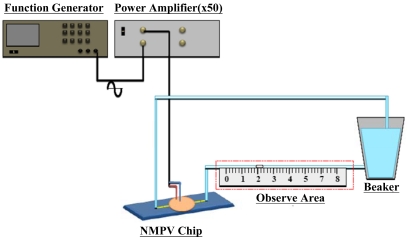
Diagram for the experiment.

Finally, the manufactured SU-8 is used as master to finish the manufacturing process of the micro-pipe to be used in the NMPV micro-pump system through the PDMS mold method. The PDMS flow channel is then bonded with glass through a hot-press process. After packaging the PZT actuator, fluid entrance/exit, and PDMS flow channel, the manufacturing of the NMPV micro-pump system is considered complete. The micro-pump system is shown in Figure 9, while Figure 10 shows a diagram of the experimental setup. A piezoelectric buzz film is used as the pressure source to drive the NMPV micro-pump system. The experiment will use a signal generator to provide the frequency required to drive the fluid. Then, the signal will be amplified by a voltage amplifier and connected to a microscope through CCD so that the movement of the bubbles in the micro-tube can be observed and images captured. The net flow is estimated according to the bubble moving distance within the micro-tube.

**Figure 11.**
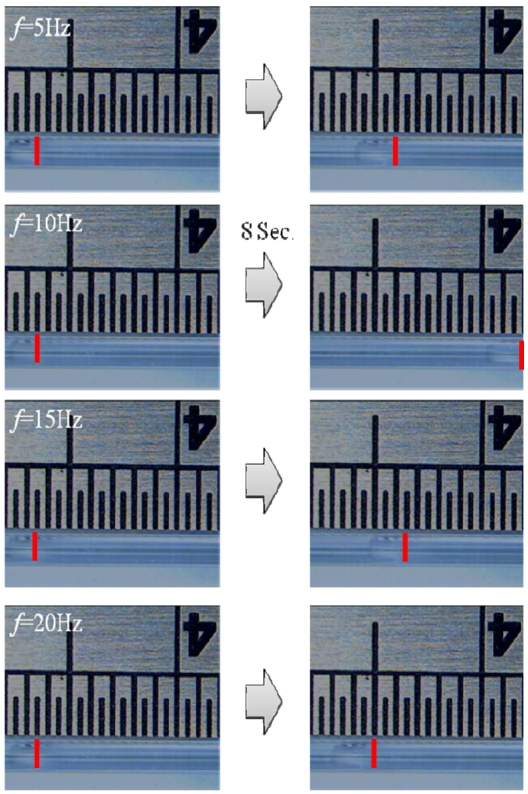
The bubble moving positions at different frequencies with an expansion valve angles of θ_1_ = 30° and θ_2_ = 120° and a driving voltage of 150 Vp-p.

Figure 11 shows the results of bubbles moving within the micro-tube under different driving frequencies at an operating voltage of 150 Vp-p. The red line represents the bubble front. When the driving frequency is *f* =5 Hz, the bubble moves roughly 2.1 mm in 10 seconds within the micro-tube. Therefore, the speed is about 6.7E-3 (μL/min.). When the driving frequency is *f* = 10 Hz, the bubble moves roughly 8 mm in 8 seconds within the micro-tube. When driving continuously for 10 seconds, the speed can be about 3.02E-2 (μL/min.). When the driving frequency is *f* = 15 Hz, the bubble moves roughly 2.5 mm in 10 seconds and the speed is about 7.71E-3 (μL/min). When the driving frequency is *f* = 20 Hz, the bubble moves roughly 1 mm in 10 seconds and the speed is about 2.38E-3 (μL/min). From the experimental results, it is found that the net flow of the NMPV micro-pump correlates positively with the vibration amplitude of the piezoelectric film measured by a Doppler laser instrument. The higher the driving voltage is, the greater the amplitude that a piezoelectric buzz film can generate and the greater the resultant net flow. Additionally, NMPV pumps work better in low driving frequencies.

**Figure 12.**
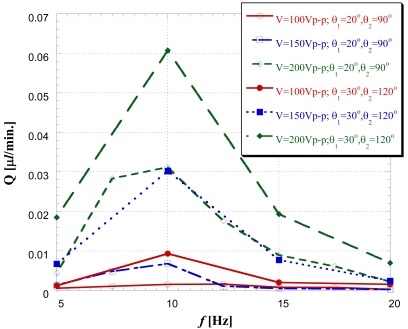
The net flow comparison chart for different driving voltages and driving frequencies with expansion valve angles of the θ_1_ = 30°, θ_2_ = 120° and θ_1_ = 20°, θ_2_ = 90°.

**Figure 13.**
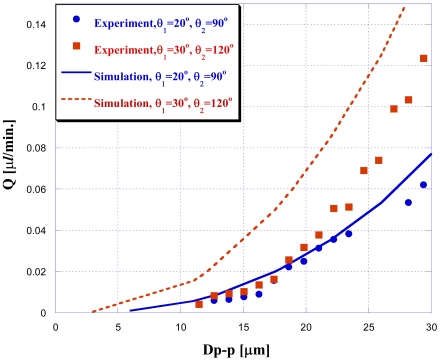
Experimental and simulation analysis comparison chart with expansion valve angles of θ_1_ = 30° and θ_2_ = 120°.

Figure 12 is the net flow comparison chart between the optimized expansion valve angles of θ1 = 30°, θ2 = 120° addressed in name of optimal case and the expansion valve angles of θ1 = 20°, θ2 = 90° for an inferior case, respectively. When changing driving frequency and voltage for buzzer, the net flow in Figure 12 shows that the driving frequency *f* = 10 Hz seems to be an optimal driving frequency because it produces a maximum net flow for the studied diffuser angle cases. In addition, it is found that the optimized design of the expansion valve of (30°, 120°) does indeed perform better at driving fluids than other studied cases. Generally speaking, the larger the driving voltage applied, the more the amount of net flow rate would be. As for the cause of the large deviation between simulations and experiments in Figure 13: the mesh independent test had been executed before the simulations. For the boundary conditions, the concerned boundary effect is not dominant and could be neglected in the numerical simulations. Oppositely, the reasons for the deviation between the experiment and simulation are mainly a result of using a bubble in the channel when the net mass flow rate with respect to the bubble moving distance is determined (this was not addressed in the simulation). In addition, the inlet/outlet port of the pumping chip was connected with an extended duct to connect the tank but this was also absent in the simulation. Overall, the optimized design of the expansion valve does indeed perform better at driving fluids than other studied case.

## 6. Conclusions

The results obtained from our simulation and experimental analyses can be summarized as follows:

1. There exists a better operating area, 1.02 < D*^p^,_i_* < 1.14, with D = 500 μm for the expansion mouthpiece, 20 < Re_D_ < 75 for Reynolds number, and 30° < θ_1_ < 57°,110° < θ_2_ < 120°.
2. Piezoelectric buzz films work better at a driving frequency of f < 30 Hz. They will produce the greatest net flow at a driving frequency of *f* = 10 Hz,
3. The produced net flow is largest when the expansion valve is D = 500 μm and its expansion valve angles are θ_1_ = 30° and θ_2_ = 120°, which are also the best angles.
